# Not living with both parents is associated with more health- and developmental problems in infants aged 7 to 11 months: a cross sectional study

**DOI:** 10.1186/s12889-015-1505-z

**Published:** 2015-02-19

**Authors:** Nadine Kacenelenbogen, Michèle Dramaix-Wilmet, Marco Schetgen, Michel Roland

**Affiliations:** Département de Médecine Générale, Université Libre de Bruxelles, Campus Facultaire Erasme, Route de Lennik 808/612, 1070 Bruxelles, Belgium; Centre de Recherche en Epidémiologie, Biostatistique et Recherche Clinique, Ecole de santé publique, Université Libre de Bruxelles, Campus Erasme CP598, Route de Lennik 808, 1070 Bruxelles, Belgium

## Abstract

**Background:**

In Western countries, many children are affected by the separation of their parents. Our main objective was to assess the possible impact of parental separation family structure on certain aspects of somatic health in low-age children.

**Methods:**

We conducted a cross-sectional study based on data collected in the framework of free preventive medicine consultations in the French Community of Belgium. The data was derived from assessments conducted, between 2006 and 2012, on children 7 to 11 months after birth during which information of 79701 infants was collected regarding the risk of sudden infant death, psychomotor development, and development in terms of height and weight. The main outcome measures were: episode of risk of sudden infant death, polysomnography, home monitoring, psychomotor development, and body mass index.

**Results:**

The parents of 6.6% of the infants were separated. We established multivariable models, based on the presence or absence of confounders. The adjusted ORs (95% CI) of symptoms perceived as frightening, notably at night, of a prescription for a polysomnography, of an abnormal polysomnography result, and of follow-up by home monitoring were thus respectively **1.3** (1.1–1.6), **1.1** (0.9–1.3), **1.8** (1.3–2.4), and **1.3** (1.1–1.6). The adjusted ORs (95% CI) for psychomotor delay and for a body mass index above the 97^th^ percentile were respectively **1.3 (**1.0–1.6) and **1.2** (1.1–1.3) in the event of separation.

**Conclusions:**

This study confirms the possibility that not living with both parents is an independent risk factor for the somatic health and psychomotor development of infants. This observation should be verified because it would have a major impact on the actions of family doctors and other first-line healthcare providers, in particular with regard to information and targeted prevention.

## Background

In Western countries, many children are affected by the separation of their parents: 30 to 40% in the United States in 2011 [1] and 25% in Canada in 2001 [2]. The situation is similar in Europe [3]. In 2010 in France, 3 million children were living with one parent [4], while this affected 35% of the pediatric population in Great Britain in 2002 [5]. In Belgium in 2002, 20% of children under 16 years of age were living in a single parent or blended family [6]. Again in Belgium in 2011, there were 67 separations for every 100 marriages, and it is currently estimated that 500 000 minors are affected [7]. Certain studies have investigated the influence of parental separation on children on a somatic, psychological, and behavioral level. A nationwide survey in the United States in 102 000 families between 2002 and 2003 showed that, after adjusting for socioeconomic status, young people living in single parent or blended families suffered more often from oral, respiratory, or trauma-related problems significantly. What is more, these young people developed more adjustment disorders and difficulties at school, and required more specialized care [1]. In Denmark, a national study in a cohort that comprised children aged 0 to 15 years born between 1977 and 2004 observed an association between the experiencing of parental divorce and severe infection compared with a control group [8]. A longitudinal follow-up over several decades of a cohort of 17 000 live-born children in Great Britain in 1958 observed that parental separation inhibited the children’s growth, in particular for boys when the event occurred between 4 and 7 years of age [9]. Studies often assess the consequences of separation on older children or adults who have experienced parental separation in their past, such as an American investigation showing the link between experiencing parental separation and beginning to drink alcohol prematurely (<14 years of age) [10]. Another American study in nearly 7000 adults observed an association between the experiencing of parental separation and an increased risk of premature cardiovascular disease, of a lower level of education, of depression, and of behavior that poses a higher risk for health [11]. The same type of results in Germany is to be found [12]. An association has been observed as well between the experiencing of stressful events when young, in particular parental separation, and autoimmune diseases [13]. Finally, following parental separation, Belgian family doctors have observed somatic and psychobehavioral repercussions and they experienced difficulties in following up children with respect to chronic pathologies and adherence to the vaccination schedule, among other things [14]. However, in the literature, there were no studies found evaluating the impact of parental separation on the health of young children. Our main objective was to assess the possible impact of not living with both parents on certain aspects of somatic health (sleeping disturbances, psychomotor development, and body mass index [BMI]) in a cohort of small children between 7 and 11 months of age. The secondary objective was to identify the other factors associated with these same health problems in infants aged 7 to 11 months and usable in first-line medicine.

## Methods

### Study population

In the French community of Belgium (Wallonia –Brussels region), the Office de la Naissance et de l’Enfance *(ONE, the Office of Birth and Childhood)* [15] is the only organization that offers a collectively-structured clinical preventive service to children aged 0 to 6 years. Similar structures exist in the Flemish- and German-speaking parts of the country. These structures play a major role in the field of health promotion and primary prevention, including vaccinations for preschool children. In addition, they are in charge not only of the follow-up of children living under precarized conditions or at risk of child abuse but also of the control of adoption procedures. Children aged 7 years or more are then being taken care of by the School Medicine discipline. Therefore, in the French Community of Belgium, the ONE offers a free preventive check-up program from pregnancy up to 6 years of age, with the data being centralized in a computerized databank. The data is collected for very young infants at five points in time: at birth in the maternity hospital, after the return home, between 7 and 11 months, between 16 and 20 months, and between 28 and 32 months. For each point of this check-up program, a data collection sheet is completed by a nurse, midwife, social worker, pediatric doctor, or family doctor who is specifically trained for this task. Once completed, these sheets are anonymized and encoded in the central database. This system is in place for evaluation purposes and facilitates the adapting of policy in the area of perinatal and early childhood social medicine. We analyzed the data of 79 701 infants who were entered in the ONE database between 2006 and 2012 and for whom there was a preventive health assessment 7 to 11 months after birth.

### Assessment of main exposure

Family structure came under six categories: parents together, parents separated, the child only sees one parent, the child is in a children’s home/home/foster home, other situations (grandparents, other parents), and unknown. For our analyses, only non-separated and separated parents (n = 78 008) were included, with children who only see one parent falling under the second category.

### Assessment of other covariates

The other independent variables included in the analyses were the age of the mothers at childbirth, their standard of French, their level of education, and their occupation. Maternal age was categorized by separating very young mothers (<18) from older mothers (≥38, the age at which amniocentesis is automatically recommended). The corresponding paternal characteristics were not taken into account because they very closely correlated with those of the mother and were only available between 2010 and 2012. What is more, a large quantity of data was missing due to the overrepresentation of “unknown” answers. For the study of variables concerning the risk of sudden infant death syndrome (SIDS), we have also taken into account breastfeeding behavior, gender and birth weight of the infants, as well as tobacco smoking within the home. The use of the binary variable regarding exposure to smoking forced us to divide our sample according to two separate periods (2006–2009 and 2010–2012), given that the wording of the question was different, which did not pose a problem given the size of the sample. The “unknown” answers were eliminated from the analyses. However, we observed beforehand that the distribution of the variables of socioeconomic status did not significantly differ between the “unknown” and included groups. For the multivariable analyses, the categories of the independent variables were grouped according to the categories presented in the tables.

### Outcome ascertainment

The dependent binary variables that were initially considered based on our research question were the risk of sudden death infant syndrome (risk of SIDS), the prescription of a polysomnography and its result, the fact that the child underwent home monitoring, as well as the child’s psychomotor development and BMI. With regard to the variables relating to the risk of SIDS and to the prescription of a polysomnography and its result, analysis was only carried out for the 2006–2009 period; from 2010 onward, only the variable regarding home monitoring remained. This did not pose a problem given the size of the sample. For the variable relating to the risk of SIDS*,* the person in charge of the assessment was asked to evaluate this risk while referring to the alarm signs listed in the ONE guidelines on preventive medicine [16] (Table 1). This list was submitted to the parent, who was then asked whether the child had presented with these symptoms or not. The presence of a sole symptom was sufficient to allow the answer to the question to be ticked as “yes”. Yet we are not in the position to focus on the different items given that the question was presented as follows: *The child has presented a risk of sudden death infant syndrome: yes/no/unknown*. We acknowledge that the list of alarming symptoms selected by the ONE staff and handed over to the parent, was larger than the classical list pertaining to the risk of SIDS or Apparent Life-Threatening Event (ALTE) [17]. For this reason, we now propose the wording «Child symptoms perceived as frightening». Home monitoring means that a specialized medical team had previously suspected an increased risk of sudden infant death requiring further examinations like polysomnography that turned out positive. The variable evaluating psychomotor development determined whether at least two abnormalities were or were not present at the time of assessment. The health professionals in charge of preventive screening evaluate the child’s psychomotor development according to his age in months, taking into account the child development stages [16]. The BMI variable was calculated based on height and weight according to the usual formula (weight in kg/height in m^2^). By taking growth charts into account (WHO 2006), we determined whether children had a normal BMI-for-age (≥3rd percentile and ≤97^th^ percentile) or not (<3rd percentile and >97th percentile).

**Table 1:** **Frightening symptoms listed in the ONE guidelines on preventive medicine**

**General symptoms**	Rectal hyper-/hypothermia
	Behavioral changes
	Turning pale or blue or losing consciousness when crying
	Becoming floppy and no longer eating or dozing
	Unpleasant odor
	Suffers a fall
**Feeding problems**	Coughing/choking/breathing difficulties while suckling
	Regurgitation long after suckling
	More liquid and more frequent stools
**Signs during sleep**	Turns pale or blue
	Respiratory arrest or frequent pauses or breathing difficulties
	Havy sweating
	Unpleasant smelling sweat
	Noisy breathing or snoring without having a cold

### Statistical analysis

The chi^2^ test was applied and the odds ratios along with their confidence interval at 95% (95% CI) were derived to compare the two groups of infants aged 7 to 11 months (exposed/not exposed to “not living with both parents”). Considering our outcomes, we are convinced that three of them (psychomotor development, BMI > p97, and BMI < p3) represent parameters that are posterior to parental separation, although we do not know the time interval in between. For these three items, we were able to confirm that logistic regression (leading to Odds Ratio) achieved similar results to complementary log-log regression analysis (leading to Rate Ratio). For the four other outcomes (child symptoms perceived as frightening, polysomnography ordered, polysomnography abnormal, and home monitoring), our transversal study did not allow us to know whether parental separation was anterior or posterior to these events. Based on this observation, logistic regression was maintained for all the models. Yet, taking into account the low outcome rates, we replaced the term “odds” by “risk” in our study in order to facilitate the understanding. We established multivariable models relying on the presence or absence of confounders. The potential confounders were as follows: age, gender, birth weight, mother’s French and educational level, tobacco exposure (only between 2006 and 2009), breastfeeding status, and mother’s age.

For our analysis, we have considered a relative difference threshold of 10% to define confounders. In order to identify confounders, we have worked on subgroups of complete cases (outcome, couple, and potential confounders). Complete cases were compared to incomplete ones with respect to the potential confounders considered (57290 children with values for all confounders and valid value for parental status and 22411 children with at least one of these variables missing). The numbers of subjects for each model (Table 2) are different because of missing values for the dependent variable. The comparison of complete cases and incomplete ones for the identified confounders did not reveal any major difference in terms of confounders and outcomes, with the exception of the mother’s French level, where there was understandably much more missing data. To work on complete cases should thus not majorly impact our study conclusions. For each model, we have then calculated the attributable risk. Interactions between the family structure and the other predictors were tested. No significant interaction was found except for the case of BMI > p97, for which stratified analysis of the mothers’ level of education proved to be necessary. To confirm that the models were adequate, the Pearson goodness-fit-test (GOF) was used, since with a number of possible groups fewer than 6, the Hosmer–Lemeshow test has low power [18]. Regarding the potential association between other confounders and the outcomes, the chi^2^ test was applied while working on the same subgroups of complete cases (Table 3). The analyses were conducted using the STATA 12.0 software (http://www.stata.com).

**Table 2:** **Description of study population**

	**Complete cases** ^**[a]**^	**Incomplete cases** ^**[a]**^	
**Variable**	**% (n)**	**% (n)**	**P-value** ^**[b]**^
*Gender*			**0.30**
Male	**50.4 (28897)**	**50.9 (10898)**	
Female	**49.6 (28393)**	**49.2 (10532)**	
*Child’age (months)*			**<0.001**
6-8	**45.8 (26232)**	**44.9 (9257)**	
9-10	**51.6 (29556)**	**51.9 (10694)**	
11-12	**2.6 (1502)**	**3.2 (651)**	
*Birth weight*			**0.02**
<2500 g	**6.8 (3880)**	**7.3 (1529)**	
≥ 2500 g	**93.2 (53410)**	**92.7 (19569)**	
*Mother’s age at childbirth in years*			**<0.001**
< 18	**1.02 (586)**	**1.16 (239)**	
18/37	**92.4 (52932)**	**91.4 (18899)**	
≥38	**6.6 (3772)**	**7.5 (1551)**	
*Mother’s level of education*			**<0.001**
< lower secondary education	**8.9 (5127)**	**11.3 (930)**	
Complete lower secondary education	**18.1 (10382)**	**16.5 (1355)**	
Complete upper secondary education	**34.7 (19861)**	**30.4 (2488)**	
Complete higher education/academic or not	**38.3 (21920)**	**41.7 (3419)**	
*Mother’s French language level*			**<0.001**
None	**2.9 (1672)**	**5.8 (1186)**	
Basic	**5.6 (3192)**	**10.4 (2129)**	
Proficient	**91.5 (52426)**	**83.8 (17093)**	
*Exclusive breastfeeding*			**<0.001**
Never breastfed	**24.9 (14245)**	**28.1 (5349)**	
Breastfed for some time	**70.1 (40159)**	**65.7 (12522)**	
Still breastfed	**5.0 (2886)**	**6.2 (1183)**	
*Smoking daily in the house (2006-2009)*			**<0.001**
Yes	**21.9 (6328)**	**24.0 (2522)**	
No	**78.1 (22567)**	**76.0 (7981)**	
*Family structure*			
Separated parents/sees only one parent	**6.6 (3370)**	**6.6 (1373)**	
Parents together	**93.4 (53520)**	**93.4 (19345**	**0.8**

^[a]^Complete cases = cases with values for all potential confounders listed in the table except smoking daily in the house available only for a sub-sample; incomplete cases = cases with at least one confounders missing.

^[b]^Test of homogeneity of proportions.

**Table 3:** **Risk of sudden death, psychomotor development, and BMI - infants aged 7 to 11 months according socioeconomic factors and children’s characteristics**

	**Frightening symptoms (2006-2009) n = 28174**	**Polysomno-graphy ordered (2006-2009) n = 30675**	**Abnormal polysomno-graphy (2006-2009) n = 2406**	**Home monitoring (2006-12) n = 49275**	**Psychomotor delay (2006-2012) n = 55180**	**BMI < p3 / ≥ p3- ≤97 (thinness) (2006-2012) n = 50810**	**BMI > 97 / ≥ p3- ≤97 (overweight) (2006-2012) n = 53978**
**Variables**	**%**	**%**	**%**	**%**	**%**	**%**	**%**
*Mother’s age at birth (years)*							
≥ 38	5.3	10.9	9.4	3.5	1.7	0.9	8.8
18-37	4.3	8.8	21.8	2.9	1.6	0.9	6.7
< 18	6.3	9.1	25.3	3.6	2.1	1.2	7.5
P-value^[a]^	0.06	0.6	0.2	0.08	0.583	0.3	0.03
*Mother’s level of education*							
Higher education	3.7	8.8	22.8	3.3	1.4	1.0	4.6
Upper secondary education	1.4	9.1	20.4	2.9	1.5	0.9	7.0
Lower secondary education	5.4	9.4	21.9	3.0	1.7	0.7	8.8
< lower secondary education	5.0	7.0	26.8	3.0	2.6	1.0	10.7
P-value^[a]^	< 0.001	0.02	0.3	0.9	< 0.001	0.1	< 0.001
*Mother’s French language level*							
Proficient	4.6	9.5	21.8	3.1	1.6	0.9	6.4
Basic	2.2	2.7	28.3	1.3	1.4	0.4	10.9
None	2.7	1.6	44.4	1.3	2.3	0.9	10.2
P-value^[a]^	< 0.001	< 0.001	0.2	< 0.001	0.067	0.01	< 0.001
*Child’s gender*							
Female	4.2	8.6	20.4	2.8	1.5	1.1	5.5
Male	4.6	9.2	23.2	3.1	1.8	0.7	7.9
P-value^[a]^	0.1	0.6	0.09	0.06	0.004	< 0.001	< 0.001
*Child’s age (months)*							
6-8	4.3	8.8	21.4	2.9	1.4	0.9	6.5
9-10	4.4	9.0	22.1	3.0	1.8	0.9	6.9
11-12	4.1	9.1	24.1	3.4	2.6	1.2	6.8
P-value^[a]^	0.8	0.8	0.8	0.3	<0.001	0.3	0.2
*Birth weight*							
≥2500 g	3.2	7.4	19.1	2.1	1.4	0.8	6.9
<2500 g	21.2	29.5	32.2	14.4	4.0	2.3	4.6
P-value^[a]^	< 0.001	< 0.001	< 0.001	< 0.001	< 0.001	< 0.001	< 0.001
*Exclusive Breastfeeding*							
Some time and still breastfed	2.8	4.9	31.2	2.2	1.4	0.8	9.9
Some time	3.8	8.2	21.1	2.6	1.4	0.9	6.5
Never	6.3	11.6	22.6	4.2	2.2	1.0	6.8
P-value^[a]^	< 0.001	< 0.001	0.1	< 0.001	< 0.001	0.3	< 0.001
*Smoking in the house (* ***2006-09*** *)*	(n = 28147)	(n = 27547)	(n = 2167)	(22868)	(n = 27962)	(n = 25693)	(n = 27310)
No	3.8	8.4	20.8	2.8	1.4	0.9	6.4
Yes	6.2	10.4	21.9	3.8	2.0	0.9	8.1
P-value^[a]^	< 0.001	< 0.001	0.6	< 0.001	<0.001	1.0	< 0.001

^[a]^Test of homogeneity of proportions.

### Ethics approval

The research protocol was approved by the local ethics committee (ERASME hospital; medical board’s approval number: OM 021) on January 24, 2012 under the following reference: P2012/026 (See attached document). The study data originated from a database that was anonymised; the ethics committee deemed that it was impossible and not necessary to obtain the informed consent from participants or their representative.

## Results

In our sample, we counted a few more boys than girls, and the proportion of infants with a very low birth weight (<2000 g) was close to 2%. Nearly 40% of mothers were aged between 25 and 30, around a third held a higher education degree, and for a little less than half of them, their main occupation was taking care of their children and the home or they were unemployed. One mother in 10 could not speak French properly, and nearly 3% of the families did not have a steady income (Table 2). Regarding parental behavior, one child out of every four had never been breastfed, and 20% were exposed to tobacco smoking between 2006 and 2009, as against 11% between 2010 and 2012 (Table 2). Across the entire sample, close to 7% of the infants had separated parents or were living with only one of their parents (Table 2). Between 2006 and 2009, in the event of parental separation, more than 6% of infants displayed symptoms perceived as frightening as against 4% when the parents were together (p-value <0.001) (Table 4). During the same period, when there was parental separation, 10% of infants underwent a polysomnography as against 8% when the parental couple was intact (p-value =0.005) (Table 4). The result of this examination was abnormal in 32% of cases when the parents were separated versus 21% when the couple remained together (p-value <0.001). Between 2006 and 2012, if there was separation, 4% of infants were followed-up by home monitoring as against 3% when the parents were together, with a risk of 1.5 (95% CI: 1.2–1.8). In our sample, approximately 1.6% of infants displayed psychomotor delay if the parental couple was intact versus 2.3% when parents were separated, with a risk of 1.4 (95% CI: 1.1–1.8). There was a significant association between the infants’ being overweight (BMI > 97^th^ percentile) and parental separation, with a risk of 1.4 (95% CI: 1.2–1.6). Separation was inversely associated with the infants’ thinness (BMI < 3^rd^ percentile), though the differences did not reach statistical significance, with a risk of 0.7 (95% CI: 0.5–1.1). No confounders were found for the association between family structure and the result of the polysomnography. After adjustment (Table 4), the other ORs were generally slightly lower than the crude results and remained significant, except for ordered polysomnography. The association between family structure and the child’s follow-up in the presence of home monitoring remained significant, with a single confounder: the child’s birth weight*.* Regarding infants’ being overweight, only the level of education was recognized as confounder. The logistic model for BMI >97^th^ percentile including family structure and mother’s education level did not fit but we observed a significant interaction between these two variables: by comparison with parents living together, in case of separation, when mothers had at least completed upper secondary education, the adjusted OR of a BMI >97th percentile was 1.7 (95% CI: 1.4-2.0; P-value <0.001), whereas for lower levels of education, the association with family structure was no longer significant and the OR was 0.9 (95% CI: 0.8-1.1; P-value: 0.30). Low birth weight was the factor that was the most closely associated with the various variables relating to the risk of SIDS (symptoms perceived as frightening, polysomnography and its results, as well as home-monitoring) (Table 3). The absence of exclusive breastfeeding also increased this risk. Conversely, when mothers did not speak French fluently, this appeared to have a protective influence (Table 3). The very low level of mother’s education, low birth weight, absence of exclusive breastfeeding, and male gender were significantly associated with psychomotor delay (Table 3). Low birth weight significantly increased the risk of thinness in the infants, whereas male gender and the fact that the mother only spoke a little French appeared to be protective factors. When the infant was a boy and when the mother did not speak French, we observed a significant increase in the risk of a BMI >97^th^ percentile, whereas low birth weight and the absence of breastfeeding were inversely associated with such a BMI (Table 3).

**Table 4:** **Risk of sudden death, psychomotor development, and BMI - infants aged 7 to 11 months: crude and adjusted results**

	**Frightening symptoms (2006-2009)** ^**[a]**^ **n = 31409**	**Polysomnography ordered (2006-2009)** ^**[a]**^ **n = 30675**	**Abnormal Polysomnography (2006-2009)** ^**[b]**^ **n = 2406**	**Home monitoring (2006-12)** ^**[a]**^ **n = 49275**	**Psychomotor delay (2006-2012)** ^**[a]**^ **n = 55180**	**BMI < p3: thinness (2006-2012)** ^**[b]**^ **n = 50810**	**BMI > 97 :overweight (2006-2012)** ^**[c]**^ **n = 53978**
*Family structure*							
Parents separated	6.4% (n = 2174)	10.5% (n = 2127)	31.8% (n = 192)	4.2% (n = 3255)	2.3% (n = 3624)	0.7% (n = 3279)	8.8% (n = 3573)
Parents together	4.2% (n = 29235)	8.7% (n = 28548)	21.0% (n = 2214)	2.9% (n = 46020)	1.6% (n = 51556)	0.9 %(n = 47531)	6.6% (n = 50405)
Crude OR (IC95 %)	1.5 (1.3-1.8)	1.2 (1.1-1.4)	1.8 (1.3-2.4)	1.5 (1.2-1.8	1.4 (1.1-1.8)	0.7 (0.5-1.1)	1.4 (1.2-1.6)
P-value^[d]^	< 0.001	0.005	< 0.001	< 0.001	0.002	0.13	< 0.001
Adjusted OR (IC95%)	1.4 (1.1-1.6)	1.1(0.9-1.3)	1.8 (1.3-2.4)	1.3- (1.1-1.6)	1.3 (1.0-1.6)	0.7 (0.5-1.1)	1.2 (1.0-1.3)
P-value^[e]^	0.001	0.12	< 0.001	0.006	0.049	0.13	0.012
AR%	26 (10.7 to 38.6)	8.3 (-6.4 to 21.0)	44 (23 to 58)	22.5 (6.5 to 35.5)	21.0 (0.1 to 37.6)	−39 (-112 to 9)	14.5 (3.3-24.2)
P-value - GOF test	0.13	0.30	-	0.21	0.61	-	<0.001

^[a]^Adjusted for Birth weight.

^[b]^No confounders.

^[c]^Adjusted for mother’s education level.

^[d]^Test of homogeneity of proportions or of the hypothesis: crude OR = 1.

^[e]^Test of the hypothesis: adjusted OR = 1.

## Discussion

After adjusting for social, economic, and cultural factors as well as for the age of the mother at childbirth and other potential confounders, we observed a significant increase in the percentage of health problems in infants when the parents were separated compared with situations in which the parental couple was intact.

### Concerning the risk of SIDS and family structure in the literature

There are studies of the risk of SIDS that, on the one hand, confirm the predictors analyzed here (low birthweight, exposure to tobacco smoking, male infant, and feeding practices) and that, on the other hand, also note the link with the marital status of the mother. Of these studies [19,20] , let us take a Canadian retrospective case-control study of 1000 deaths that found using logistic regression an over-representation of newborns of non-married mothers (OR: 3.48; 95% CI: 2.94–4.11) [21]. Similarly, a Canadian prospective study that analyzed a cohort of more than 40 million infants born between 1995 and 2004 found a RR of SIDS of 1.7 (95% CI: 1.6–1.8) when the mother was single, taking mothers in a couple for reference [22]. There are also British retrospective studies that have found by means of univariate analysis an OR of SIDS of 3.00 (95% CI: 1.89–4.77) when the mother was single [23]. Yet these results cannot be compared to others: The focus has usually been on the fact that the mother was single, and not on parental separation and its possible corollary the blended family. Above all, the results described concerned cases of SIDS and not of ALTEs, for which we have not found any studies exploring a possible link with family structure. It should also be mentioned that in our study, the list of symptoms considered as frightening and sought for in the infants (Table 1) was much larger than the classical list pertaining to ALTE [17]. Furthermore, although the literature shows similarities in terms of risk factors [24], it appears that SIDS and ALTEs should not always be thought of as consequences of a single process [25]. Polysomnography is useful in very young children for detecting sleep disorders that require special care. Besides certain genetic malformations and abnormalities, the main indications for this examination are the testing for obstructive apnea, the snoring associated with nocturnal desaturation, gastroesophageal reflux, and laryngomalacia [26,27]. Our study brings to light a strong association between parental separation and abnormal polysomnography results. We cannot find any direct explanation for this observation in the literature. Investigations have linked certain nocturnal phenomena (snoring and other breathing noises, abundant sweating) to shorter sleep duration [28] or a less-advantaged socioeconomic environment [29], factors that may indirectly suggest the influence of parental behavior, which can vary depending on circumstances. For example the absence of exclusive breastfeeding is a risk factor for obstructive sleep apnea as well as for reflux and large regurgitation [30], but several literature reviews have reported the influence of marital status and of the presence of the infant’s father in decision-making with regard to breastfeeding, as well as that of the duration of breastfeeding [31,32]. In our study, the differences reported regarding breastfeeding behavior [33] cannot explain why the infants of mothers who do not speak French fluently are at a clearly lower risk of ALTE. Cultural habits relating to the sleep of infants should perhaps not be ruled out [34]. Our results did not reveal any confounder, even when taking into account the type of alimentation received (Table 4). A possible hypothesis would be the following: When the child’s parents do not live together, this would more often lead to a clinical picture meeting the criteria for reimbursement of home monitoring. Considering the transversal design of our study, we must admit that this is only a hypothesis.

### Delayed psychomotor development (PMD)

In our sample, less than 2% of the infants displayed a psychomotor delay, and the adjusted OR was 1.3 (95% CI: 1.1–1.6) if there was parental separation. The literature gives us at least three possible explanations. The first points to the influence that the quality of both parents’ involvement in the upbringing of the infants has on the infants’ cognitive-behavioral development, as several systematic reviews and meta-analyses have illustrated [35,36]. A longitudinal study in 290 infants aged 24 to 36 months showed that the father’s involvement had a direct impact on the emergence of the infants’ developmental acquisitions, in particular that of language, while also having an indirect influence by improving the mother–child connection [37]. The second possible explanation recalls that the parental couple faces difficulties in the months following childbirth that increase the risk of maternal and paternal depression [38,39] and that this depression generates developmental disorders in infants [40,41]. Furthermore, several research works have shown that children that do not live together with both parents are more likely to be victims of child maltreatment or neglect. These are circumstances under which developmental retardation among children aged 0-6 years has been more frequently observed [42,43]. A link has been documented between the separation of the couple and violence between partners [44]. In 40% of cases, infants of 7 years of age and under witness this violence, the consequence of which is adjustment disorders [45]. Family doctors have observed the same problems: namely, violence between ex-partners and developmental disorders in infants after separation [14].

### Body mass index (BMI)

In our sample, the BMI of 7% of the infants was above the 97^th^ percentile, and 1% of the infants seemed thin, with a BMI below the 3rd percentile. We did not find any truly significant association between family structure and the risk for the infant of having a BMI <3^rd^ percentile. However, when the mother had at least finished her secondary education, parental separation revealed an increased adjusted OR of 1.7 for a BMI >97^th^ percentile. It should be noted that infants attain a BMI peak at the age of 7 months, after which BMI starts to decline from the eighth month onward. In other words, attaining a BMI >97^th^ percentile at that age is possibly the beginning of an early adiposity rebound. The literature confirms that economic factors and some types of behavior (infant feeding practices, smoking tobacco during pregnancy, parental obesity, lifestyle) increase the risk of being overweight from early childhood onward [46-48]. However, no authors have directly linked family structure and early adiposity rebound. While several authors reveal an association between the parental engagement level from an educational or affective point of view and children’s overweight [49,50] or feeding practices [31,32], it is impossible as yet to confirm an explicative link between these research works and our observations.

### Strengths and limitations of this study

Regarding the main independent variable, namely the family environment, we found that less than 7% of the infants lived under parental separation by adding the “parents separated” and “infant only sees one parent” categories together. This percentage seems low given national statistics [7]. Of note is that in the French-speaking part of Belgium, the percentage of families where the two parents are separated increases with the child’s age: in 2009, 6.6% of infants aged 6 to 11 months were living in such a setting, in comparison with 9% when considering children aged 28-32 months [51]. It is thus likely that the very low age of our study population accounts for the differing rates of separated couples encountered in our sample as compared to the overall child population (all ages confounded, 0 to 18 years).

As regards our results, caution should be exercised owing to the methods employed. The cross-sectional nature of our study results in uncertainty with regard to time: Theoretically, we do not know the direction of causality between the variables, and we have no idea of the length of time the infants were exposed to parental separation, nor whether their parents were separated or not when they were born. In this context, it should be mentioned that the child’s psychomotor development, weight, and height were evaluated at the time of the assessment, and so in any case after a potential parental separation has been notified. However, some potential confounders were not available in our database, notably the child’s medical history, general health status, manner of sleeping or mother’s weight. At the preventive consultations, while the ONE agent enquires about the risk factors within the family and contributes to the safety promotion for low-age children in terms of sleeping manners, feeding, tobacco exposure, all information is not recorded in the case record forms. Although one of the strong points of our study is the size of the sample, involving nearly 80 000 subjects (20% of the population of that age in the French Community) [52], we noted some dissimilarities in comparison with the general population. For instance, we found a difference between the genders that was 1% lower than that normally observed for this age group (1.2% versus 2.2%) [53]. Similarly, very small birth weights (≤1999 g) taken together represented 1.9% in this study, as against 2.3% on a national level [54]. A possible explication is that boys and infants with a very low birth weight have higher morbidity, and it may be that they are more often followed in a specialized medical setting than in preventive consultation at the ONE. We cannot exclude the possibility that the socioeconomic circumstances mentioned may also explain these differences. Indeed, in our sample, nearly 39% of the women held a higher education degree, as against 25% generally in Belgium [55]. Moreover, in the studied population, 14% of families lived on only one (or two) replacement salary (dole or social assistance), and great caution must be exercised in comparing this data to the 20% pertaining to the 2012 general population [56]. We know that in Belgium, there exists a positive correlation between social level and general health status [57]. Given that our study sample comprised a greater proportion of better-off families as compared to the general population, it is possible that the observed differences in relation to family structure were less marked in the former than in the latter. Despite the differences cited, all the socio-cultural strata were represented in the study population, allowing us to make comparisons according to the socio-cultural level. Lastly, within our French-speaking Community of Belgium (Wallonia-Brussels Federation), there was no other access available to such a population - the least possible selected- of infants and young-aged children. What also confirms our interpretation is that this study was undertaken in response to a “clinical impression on the ground” of first-line doctors, which was later documented in a focus-group study [14] in family doctors and which is at the root of our current research question. What is more, we have seen that the Western, and in particular European, literature has reported studies (including prospective studies) whose results support this idea of the negative impact of parental separation on the health of infants. Two other strong points of this study are that we aimed at a specific age group that had been the subject of little study beforehand with regard to the impact of family structure and, furthermore, that the data was collected by pediatric healthcare professionals. We believe that these results are noteworthy, and for us this confirms the usefulness of conducting both research into other age groups and prospective studies.

## Conclusions

### Implications for first-line healthcare providers and family doctors

Our study confirms the need for preventive action in the families of very young children on the driving out of smoking and promotion of breastfeeding as well as on the safe sleep, psychomotor development, and BMI of infants, in particular for the poorest families, in which the adult members are less well informed about their own and their children's health. In this regard, the proactivity of family doctors remains essential, because almost all the families in Belgium have an appointed family doctor whom 90% of adults and 70% of children see on average four times a year. We also know that the poorer families are, the more they go to see their doctor [58]. What these results tell us is that infants aged between 7 and 11 months whose parents are separated do present more frequently frightening symptoms, especially at night, and they display psychomotor delays, a BMI >97^th^ percentile and abnormal polysomnography results that require home monitoring more often as well. Even though this study does not give any explanation about the reasons behind these observations, it makes us step back from the idea that the less-than-optimal development of the children of separated parents is due only to an economically more precarious environment. It seems worthwhile to recommend that family doctors make it standard practice to enquire about family composition when dealing with families who have an infant or infants aged under 12 months. In the event of parental separation and regardless of socioeconomic situation, the family doctor should then be even more attentive with regard to the infant’s health. Indeed, research could be started into whether there is a need to inform young couples who wish to start a family about the impact of the family environment on the infant’s health – without lecturing or preconceptions on the doctor’s part. In Belgium, it is already advised that family doctors enquire about the quality of the partners’ relationship every time they have contact with a pregnant woman or a family with a very young child or children [59]. Our results confirm the validity of this approach, which makes it possible to support couples who are often in difficulty during pregnancy and in the months that follow childbirth. It can be presumed that if the recommendations proposed are properly understood – that is, applied in a kind and understanding manner and people are not stigmatized – then the benefits, however small, will in all cases outweigh the resulting risks. This likely merits discussion within all health professions at any rate.

## Abbreviations

ALTEs: Apparent life-threatening events
BMI: Body mass index
ONE: Office de la naissance et de l’enfance, or office of birth and childhood
PMD: Delayed psychomotor development
SIDS: Sudden infant death syndrome
